# Phenology of *Drosophila* species across a temperate growing season and implications for behavior

**DOI:** 10.1371/journal.pone.0216601

**Published:** 2019-05-16

**Authors:** Jennifer M. Gleason, Paula R. Roy, Elizabeth R. Everman, Terry C. Gleason, Theodore J. Morgan

**Affiliations:** 1 Department of Ecology and Evolutionary Biology, University of Kansas, Lawrence, Kansas, United States of America; 2 Division of Biology, Kansas State University, Manhattan Kansas, United States of America; 3 Apogee Analytics, Matawan, New Jersey, United States of America; Institut Sophia Agrobiotech, FRANCE

## Abstract

*Drosophila* community composition is complex in temperate regions with different abundance of flies and species across the growing season. Monitoring *Drosophila* populations provides insights into the phenology of both native and invasive species. Over a single growing season, we collected *Drosophila* at regular intervals and determined the number of individuals of the nine species we found in Kansas, USA. Species varied in their presence and abundance through the growing season with peak diversity occurring after the highest seasonal temperatures. We developed models for the abundance of the most common species, *Drosophila melanogaster*, *D*. *simulans*, *D*. *algonquin*, and the recent invasive species, *D*. *suzukii*. These models revealed that temperature played the largest role in abundance of each species across the season. For the two most commonly studied species, *D*. *melanogaster* and *D*. *simulans*, the best models indicate shifted thermal optima compared to laboratory studies, implying that fluctuating temperature may play a greater role in the physiology and ecology of these insects than indicated by laboratory studies, and should be considered in global climate change studies.

## Introduction

Species composition in temperate regions is dynamic, changing in response to both abiotic and biotic factors over space and time [1]. Species abundance is often constrained by specific phenologies [2], instances of which have been observed in diverse groups of species and environments including zooplankton in ephemeral pools [3], bacterial communities in lakes [4], migratory birds [5], and complex insect communities [6, 7] Often seasonal phenology is driven by abiotic environmental factors including environmental stressors, which in combination can have pronounced effects on community composition [8]. However, the addition of new species via range expansion or human-facilitated introduction can also shift the community composition and produce immediate and long-term effects [9].

Given the fragile and dynamic nature of species abundance and community dynamics, understanding the dynamics of temporal changes in communities across seasons can provide insights into the biology and ecology of organisms. In addition, identifying the ecological drivers underlying changes in phenology is critical to understanding the effects of global climate change in real time [10]. Insect communities provide models for tracking phenological shifts in species abundance because their physiology is strongly influenced by thermal variation and resource availability [11–13]. One group of species that has received limited focus in the field of community ecology are the Drosophilids. *Drosophila* species have differing phenology and environmental tolerances (e.g. [14–18]). Although multiple studies focused on diverse Drosophila taxa have documented the dynamics of single species or species groups in temperate zones (e.g. [19–22]), the majority of studies of community assemblage have been conducted in the tropics (e.g. [23, 24]). In disturbed areas of the tropics, exotic species dominate and displace native species [17, 23–26], a pattern also seen in temperate zones [27]. Thus *Drosophila* can serve as indicator species for environmental disturbance with major changes in community composition across years as a signal of environmental degradation [28].

In *Drosophila*, interspecific dynamics in the temperate region have focused mostly on two cosmopolitan species, *D*. *melanogaster* and *D*. *simulans*. Patterns of abundance in nature in the Northern Hemisphere indicate that *D*. *simulans* is more common than *D*. *melanogaster* in southern communities whereas the opposite occurs in northern communities, with seasonal variation [21]. One explanation for geographically-specific distributions of *D*. *melanogaster* and *D*. *simulans* is that *D*. *melanogaster* is more desiccation resistant [29], implying a difference in response to environmental stress, because desiccation stress alone can change community composition [30].

In the state of Kansas, few collection records exist for *Drosophila* and those available all date to the 1950s or earlier [31–34]. The most extensive collections (Table 1) occurred in the fall of 1950 and 1951, primarily in Riley County (around Kansas State University) and in Douglas County (around the University of Kansas, [33]). That study found a total of 16 species, three of which were non-native cosmopolitan species (*D*. *immigrans*, *D*. *melanogaster* and *D*. *simulans*).

**Table 1:** Species collected in Kansas.

Species group	Species^1^	Percentage of collection	
		Yoshimoto 1954^2^	This Survey^2^
busckii	busckii*	3.38	0.29
funebris	funebris*	1.65	
guttifera	*guttifera*	0.08	
immigrans	immigrans*	0.16	
melanica	*melanica*	0.39	0.24
melanogaster	melanogaster*	83.12	19.96
	simulans*	0.16	57.10
	***suzukii***		11.13
obscura	*affinis*	2.90	1.69
	*algonquin*	0.24	7.31
	*athabasca*	1.02	
repleta	hydei*	2.43	1.93
	repleta*	1.33	
robusta	*robusta*	0.86	
testacea	*putrida*	1.18	
tripunctata	*tripunctata*	0.31	0.44
virilis	*americana*	0.78	

^1^Invasive species are in **bold.** Cosmopolitan species are designated with an asterisk.

^2^Numbers are the percentage of the total collection that is each species.

Two *Drosophila* species recently invaded Kansas: *Zaprionus indianus*, first noted in 2012 (S. Noh and T. Morgan, pers. comm.) and *D*. *suzukii* in 2013 [35]. Although *Zaprionus* is a different genus, the genus *Drosophila* is paraphyletic with *Zaprionus* embedded within it [36]. These species are of particular interest because, unlike most *Drosophila*, they are agricultural pests. *Zaprionus indianus* lays eggs on developing fruit, particularly on oranges, peaches and figs (reviewed in [37]). First detected in Florida in 2006 [37], the species has been moving north and westward [38]. Previously the species invaded South American in 1998, where it has received great attention because of economic crop losses there (reviewed in [39]).

*Drosophila suzukii*, a native of Asia, recently invaded North America and Europe [40, 41]. The original invasion was in California but the species jumped to Florida from where it has spread north and westward [42]. Unlike many *Drosophila* species that lay eggs on rotting fruit, *Drosophila suzukii* females lay eggs in ripening soft fruit, damaging the fruit with their serrated ovipositor [43]. Losses observed on individual fruit may be as high as 40% for blueberries, 50% for blackberries and raspberries, 33% for cherries and 20% for strawberries [44]. Thus, *D*. *suzukii* is a pest species of great economic importance.

We monitored *Drosophila* abundance across a growing season in one year (2014) at two fruit orchards in Kansas to determine 1) what species are present and if the recent invasive species (*D*. *suzukii* and *Z*. *indianus*) are established, 2) the phenology and population dynamics of each species and 3) how community composition changes over the seasons. Overall, we found a decrease in diversity compared to sampling 60 years ago and variation in seasonal abundance for individual species, as has been found in other temperate populations.

Using our data, we built a model of species abundance given weather data, predominantly temperature, for the four most abundant species. The predictions of the model, which include minimum and maximum temperatures for positive growth rates, are discussed with respect to laboratory-based measurements on species thermal tolerances. These models are among the first that are based on the ecology of *Drosophila* species.

## Materials and methods

### Collections

From late April through early November 2014 we collected flies at two locations near Topeka, KS: Rees’ Fruit Farm (39.0913163 latitude, -95.5939707 longitude) and eleven miles away, 86^th^ Street Orchard (39.202831 latitude, -95.7415270 longitude). Owners of the farms gave permission for our collections. Both sites had a mixture of soft fruits (S1 Table). Bottle traps baited with banana mash were modified from the design of Markow and O’Grady [45]. Bottles were empty 1.75 L juice containers (Simply Orange Juice Company; Apopka, FL). Three rows of seven 3/16-inch (4.76 mm) holes were burned into one side. Bait was prepared two days in advance of setting traps; each trap was baited with 1.5 mashed bananas, approximately 30 mL water, and approximately 0.5 grams of yeast placed in the bottom of the container. Collections occurred approximately weekly or biweekly (the one exception was a 28-day gap between a collection in May and the next collection in June) with traps hung from fruit trees or in a raspberry hoophouse for 2–7 days (Table 2, S2 Table). Traps were always hung in the same locations and were pooled by date and across both locations. Dates of collections were converted to day of the year using the date at which the trap was placed. Because the number of days of collection varied, in some analyses the number of flies was corrected for the trapping duration. All *Drosophila*, live and dead, were removed from bottles and keyed to species either immediately or after storage in 70% ethanol for up to 6 months. The predominant species were *D*. *simulans* and *D*. *melanogaster*. Females of these species are difficult to distinguish; thus, these females were assigned to the two species in the same proportion as that of the males that were in the collection. In some analyses, only the males were used.

**Table 2:** Summary statistics for collections.

	Mean ± SD	Min	Max
Number of species	4.21 ± 1.78	2	8
Number of individuals per day	241.77 ± 321.95	10	1400
Number of collection days	2.48 ± 1.47	2	7
Number of days between initial dates of sequential collections	9.80 ± 5.15	6	28

Traps were set out for 2–7 days from April 18, 2014 through November 2, 2014 for a total of 21 collections.

### Ecological indices

The relative abundance of a species was determined as the proportion of a collection that was the focal species. For each date, species richness (S, the number of species in the sample) was determined. We calculated the Shannon Diversity Index [46] as

$$H\;=\;-\sum_{\text{i}}^{\text{S}}p_{i}ln(p_{i})$$ (1)

where p_i_ was the proportion of species i in the sample and H was the species richness. The inverse Simpson index [47] was calculated as

$$\frac{1}{D}\;=\;\frac{1}{\sum_{i}^{S}p_{i}^{2}}$$ (2)

Evenness was calculated as

$$J^{'}\;=\;H/H_{max}$$ (3)

Where H_max_ was the maximum diversity in the sample found when all species were equally abundant (equivalent to ln(S)). All the above indices were calculated for each collection date. The mean and standard deviation over all collections were calculated as well. Evenness was also calculated as a corrected index in which S was the total number of species found across the entire collecting season (nine species).

### Weather data

Hourly weather data for Topeka, KS were obtained from the Weather Data Library in the Department of Agronomy, Kansas State University. Specifically, hourly measurements were recorded at the Topeka Billard Municipal Airport (KTOP: 39.07 latitude, -95.62 longitude). These measurements were point estimates and not hourly means.

### Modeling the capture of flies during the collection season

We modeled the variation in the number of flies captured in the traps using the environmental variables of temperature and relative humidity. We limited our examination to the four most abundant species (*D*. *simulans*, *D*. *melanogaster*, *D*. *algonquin* and *D*. *suzukii*) because only these species provided sufficient counts to be adequately modeled. Because the basic data for each species consisted of the number of flies captured during each collection period, the natural distribution for describing the probability of a fly being captured is given by the Poisson distribution [48]:

$$Pr\;(n|\varphi )\;=\;\frac{\varphi^{n}}{n!}e^{-\varphi }$$ (4)

where n is the number of flies captures and φ is the intensity parameter (or relative abundance) of flies of a given species. The intensity parameter depends most heavily on two characteristics of the species of interest: the size of the population in the neighborhood of the traps and the level of activity of the flies while the traps are available. We hypothesized that both of these characteristics are strongly affected by the temperature and relative humidity in the region of the traps. In particular, we conjectured that the intensity parameter changes over time in a manner given by

$${\varphi_{t}\;=\;\varphi }_{t-1}e^{G_{t}}$$ (5)

Where t is a moment in time and G_t_ is a growth factor (or rate) that depends on temperature and relative humidity by the expression

$$G_{t}\;=\;A+BT_{t}+CT_{t}^{2}+DH_{t}$$ (6)

where T is temperature and H is relative humidity.

If we set φ_0_ to be the relative intensity parameter at the start of the season, then these three equations allowed us to calculate the probability of capturing n_t_ flies at any time t during the collecting season using just five free parameters: φ_0_, the intensity parameter at the start of the season, and A, B, C, and D, the constants of the growth rate. Estimates for the parameters were obtained by choosing those values that maximize the natural log likelihood of the observed data given the parameters. The likelihood of the data in these instances was the product of the probabilities of the number of captured flies in each collecting period.

Because the temperature and humidity data were measured hourly, the natural unit for the model was an hour. Collections, however, were made for longer periods, usually for 48 hours, although some collections were as long as a week. The Poisson process has the property that if intensity parameter estimates are calculated for each hour, the probabilities for an entire collection period could be calculated by simply summing the intensity parameters for each hour of that period [49]. To have a uniform starting point for all models, t = 0, we set the beginning of each model at sunrise on Day 98 of the calendar year, 10 days before the first traps were put out on Day 108.

Model selection revealed that only the daylight hours were relevant for calculating changes in the population sizes of the species. By using only daylight hours for adjusting population sizes, we effectively assumed that no flies were captured at night and no significant changes in the fly populations occurred during the nighttime hours. To test this assumption, we estimated the model parameters using all the hours of the day. These models were sensitive to average daily temperatures and not to humidity. Furthermore, the more complex models lost the ability to reflect the detailed variation in the capture data among collection periods. These models were not particularly good at fitting the data.

Once the free parameters were estimated, we assessed the quality of the models by correlating the mean number of flies captured per hour with the mean intensity parameter for each capture period. The conventional F-test for these correlations,

$$F\;=\;\frac{r^{2}(N-K)}{(1-r^{2})K}$$ (7)

where r is the correlation, N is the number of collection periods, and K is the number of parameters), yielded *P*-values less than 0.01 for all the models. All analyses were conducted with custom written scripts in R [50].

## Results

### Species composition and relative abundance

Among the 10,638 individuals identified as *Drosophila* over 21 collections, we found nine species, including three invasive species (*D*. *melanogaster*, *D*. *simulans* and *D*. *suzukii*) and six native species (Table 1). Notably absent across the entire season was *Zaprionus indianus*, which was found in Kansas at these same sites in 2012 and 2013 (S. Noh and TJ Morgan, unpublished). All the invasive species, with the exception of *D*. *suzukii*, are cosmopolitan and have been established for many years. Of the nine species found in 2014, eight were found in the state over 60 years ago in the last collection records that we were able to find for Kansas [34]. The one species found only in our collection is *D*. *suzukii*, a recent invasive species, which was first collected in Kansas in 2013 [35].

**Fig 1:**
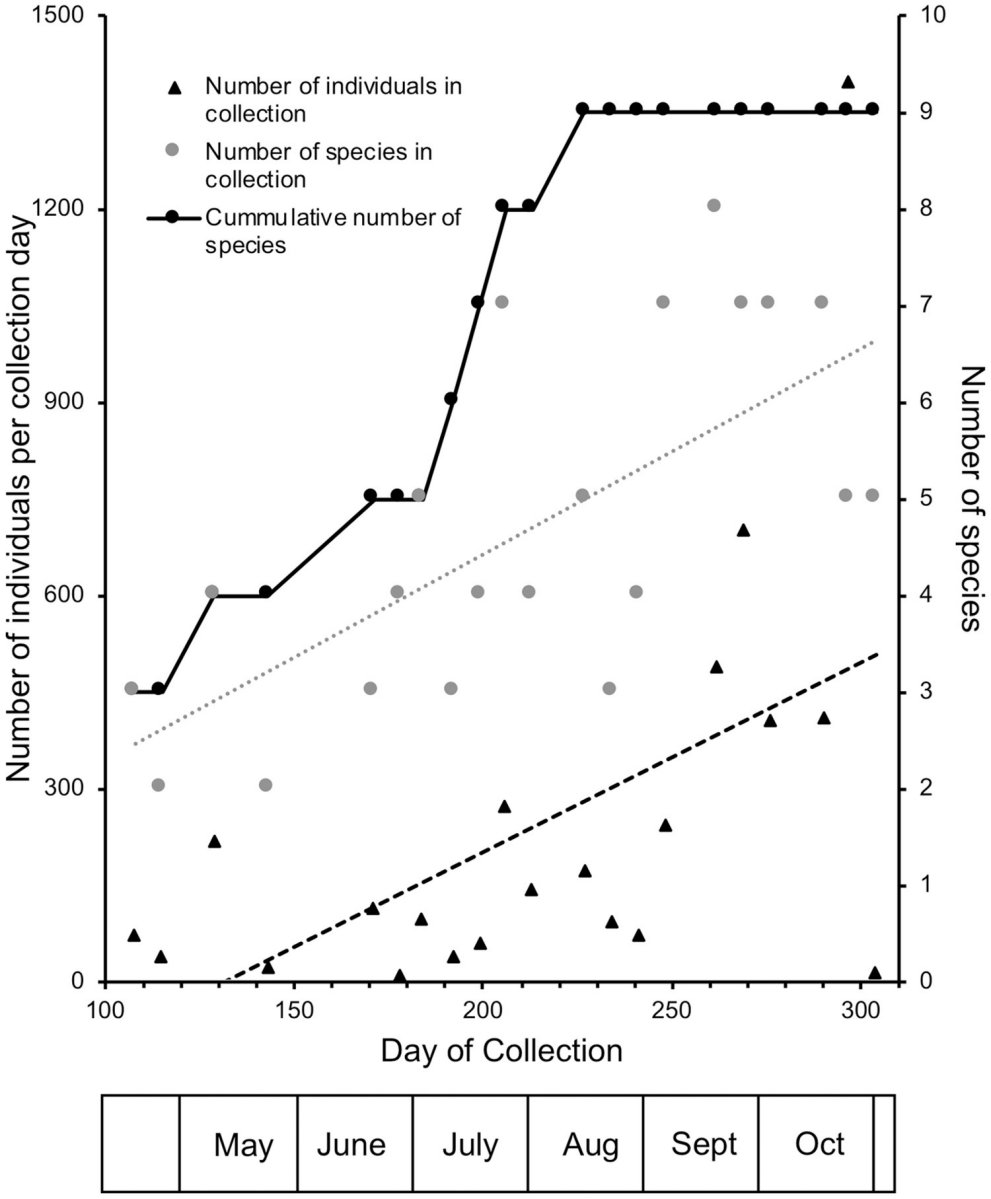
Collection statistics for the season. The cumulative number of species (black circles) reached a maximum of 9 in the August 15 (day 227) collection. The number of species in a collection (gray circles) hit a maximum in the September 19 (day 262) collection. The total number of individuals per day of collection (triangles) peaked in the September 26 (day 269) collection. The number of species caught increased with time (r^2^ = 0.483, *P* = 0.0005). Over the entire season, the number of individuals caught per collection day increased until the last collection, which coincided with the first frost (r^2^ = 0.296, *P* = 0.01).

Both the number of species and individual flies increased across the season, with different species combinations detected in different collections. Total counts of individuals per day in a collection peaked in the October 24 collection (day 297; Fig 1). The first frost in 2014 was on October 31 (day 304); that collection was greatly reduced. All collections contained at least one species with a mean of 4.21 ± 1.78 (standard deviation) species per collection day (Table 2).

**Fig 2:**
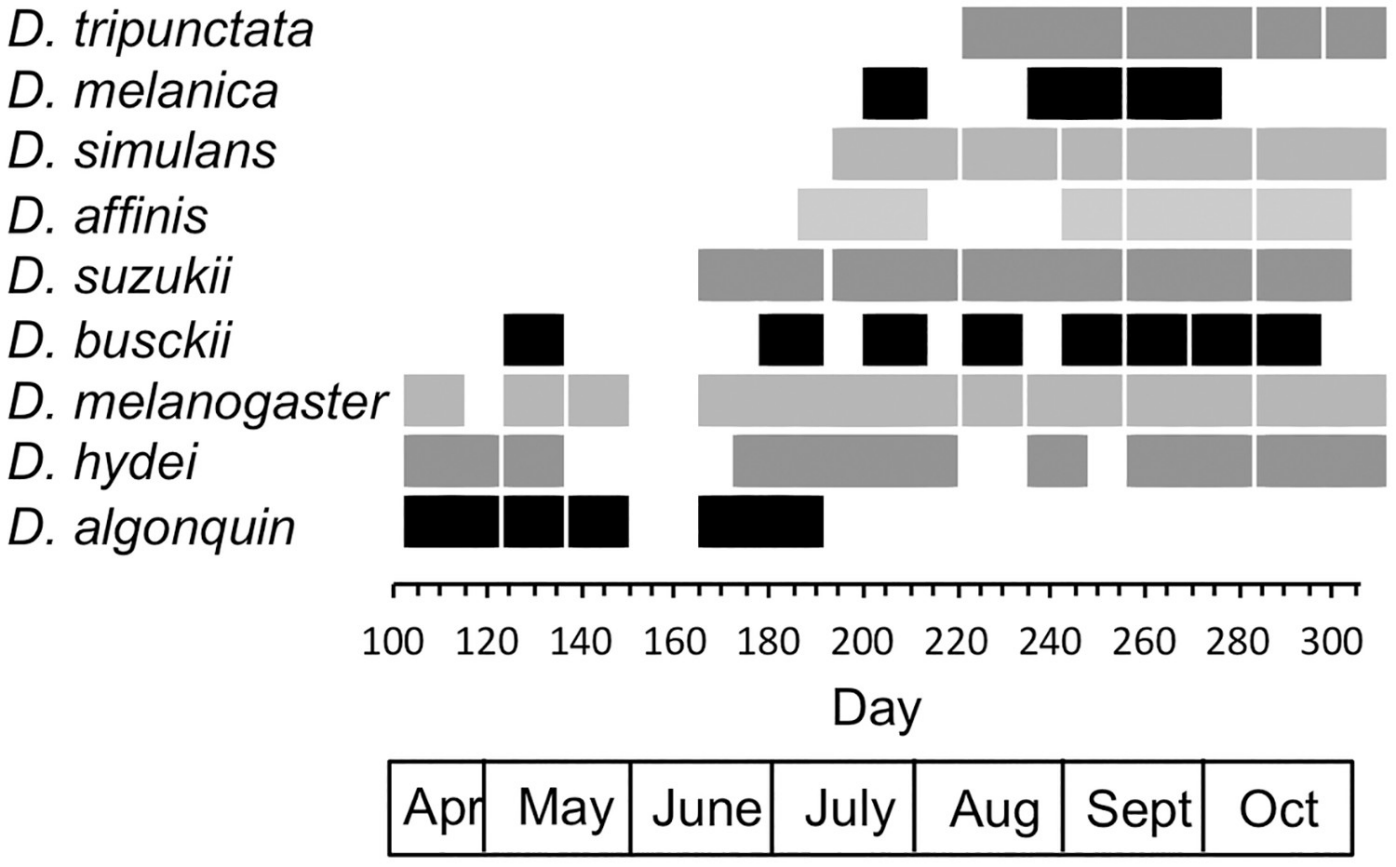
Presence and absence of each species across the season. Gaps across all species reflect gaps in collections. Numbers across the bottom reflect the day of year.

The earliest species were *D*. *algonquin*, *D*. *melanogaster* and *D*. *hydei;* the latter two species persisted throughout the collections though *D*. *algonquin* was last collected on day 184. Notably, *D*. *algonquin* and *D*. *affinis*, which are members of the same species subgroup, were never in the collection at the same time because *D*. *affinis* did not appear until day 192 (Fig 2, S3 Table), thus no single collection contained all species (Fig 1, S1 Fig). The last species to appear, *D*. *tripunctata*, was first collected on day 227. Most species were present late in the season so that one collection in September (day 262; Fig 2, S2 Table) contained the most species, eight; the missing species was *D*. *algonquin*.

**Fig 3:**
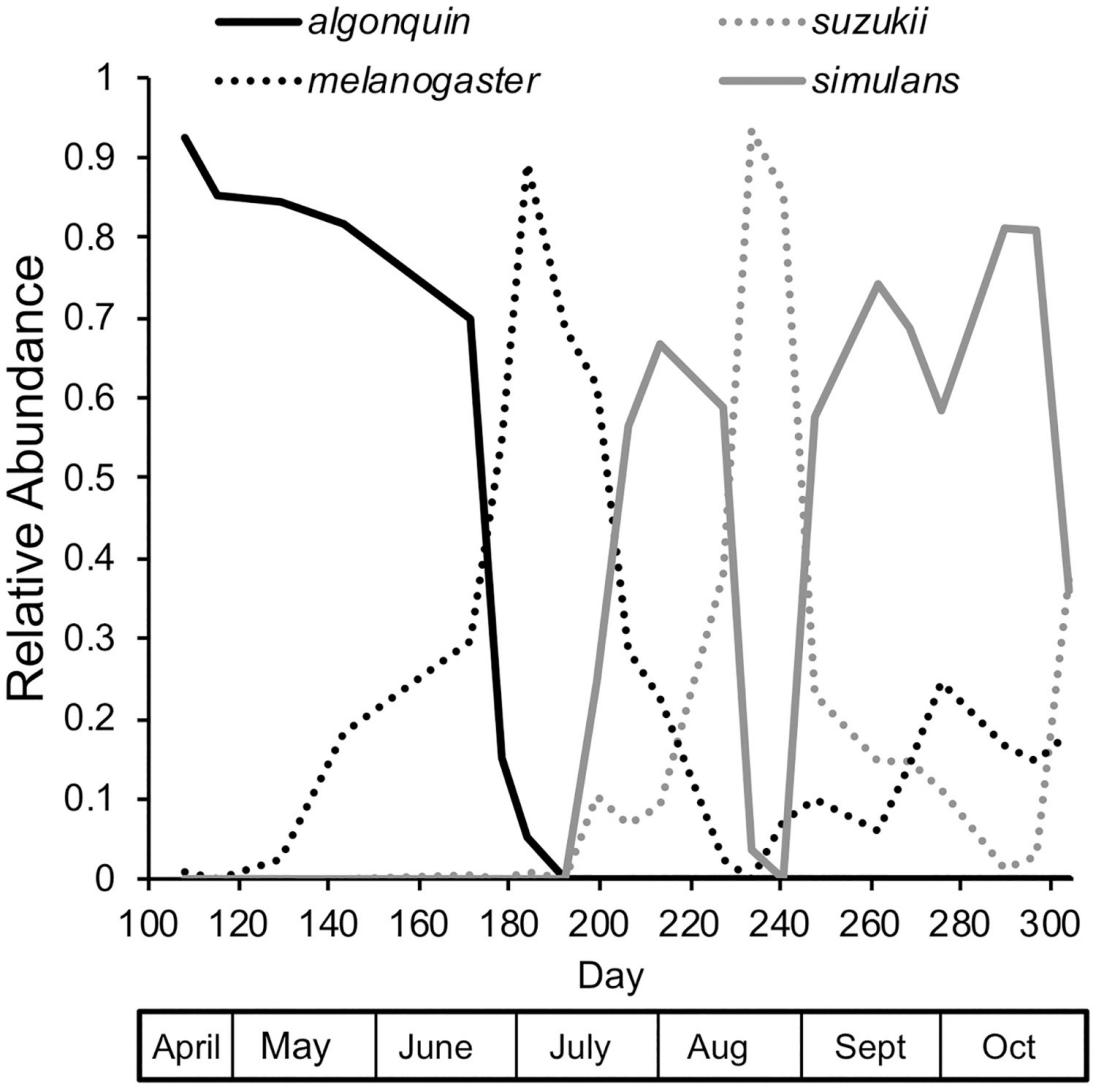
The relative abundance of the four most abundant species over the entire collection season. Only species that were 20% or more of a single collection are included.

The number of individuals of a species was variable throughout the collection period (S4 Table, S1 Fig). All species had dramatic fluctuations in the total number of individuals per collection day, with regular peaks and crashes in population size. Not all of these fluctuations covaried, thus many peaks and crashes were species-specific (Table 3), though only the most abundant species had enough individuals to find correlations. *D*. *algonquin* and *D*. *hydei*, as early season species, peaked in May (day 129) long before several of the other species appear, thus their abundance positively covaried, although *D*. *algonquin* negatively covaried with all the other species (Table 3). Unlike *D*. *algonquin*, *D*. *hydei* reappeared throughout the summer with a second, smaller peak in late October. The peak numbers of individuals collected differed greatly among species with *D*. *busckii* and *D*. *tripunctata* on the low end with a maximum of 6.5 individuals per collection day compared to the most abundant species, *D*. *simulans*, with a maximum of 1129.5 individuals per collection day (S1 Fig). At any time, one of four species, *D*. *algonquin*, *D*. *melanogaster*, *D*. *simulans*, and *D*. *suzukii* dominated the collections (Fig 3). The sister species *D*. *melanogaster* and *D*. *simulans* were the most common species in the entire collection, with *D*. *melanogaster* much more abundant than *D*. *simulans* until day 206, at which point *D*. *simulans* became more abundant than its sister species (though *D*. *suzukii* was the most abundant on that date). At the end of the season, *D*. *simulans* was far more abundant than *D*. *melanogaster*. The abundance of the three invasive species, *D*. *melanogaster*, *D*. *simulans* and *D*. *suzukii*, positively covaried; *D*. *simulans* abundance was significantly correlated with that of the other two species (Table 3).

**Table 3:** Covariance^a^ and correlation^b^ of Pair-wise species abundance^c^.

	*D*. *affinis*	*D*. *algonquin*	*D*. *busckii*	*D*. *hydei*	*D*. *melanica*	*D*. *melanogaster*	*D*. *simulans*	*D*. *suzukii*	*D*. *tripunctata*
*D*. *affinis*		-313.052	18.676	34.762	8.838	455.015	1399.066	207.631	18.838
*D*. *algonquin*	-0.521		-19.924	743.512	-48.162	-2896.985	-11468.534	-2184.369	-87.062
*D*. *busckii*	0.450	-0.105		-3.781	-0.119	57.813	142.046	20.560	3.631
*D*. *hydei*	0.195	0.160	-0.077		-9.240	511.724593	1218.320	-273.705	-19.840
*D*. *melanica*	0.484	-0.381	0.238	-0.228		-18.221	153.776	99.105	8.540
*D*. *melanogaster*	*0*.*563*	-0.377	0.174	0.417	0.137		43640.729	858.027	-65.420
*D*. *simulans*	*0*.*615*	**-0.711**	0.336	-0.003	0.376	0.505		13684.671	205.175
*D*. *suzukii*	0.317	**-0.734**	0.131	-0.368	0.566	0.229	**0.706**		153.605
*D*. *tripunctata*	0.345	*-0*.*5678*	0.323	-0.562	0.571	-0.083	0.481	**0.745**	

^a^Covariances are in the upper diagonal

^b^Spearman correlations are in the lower diagonal

^c^Values in bold: ***P* < 0.0014** (Bonferroni correction for 36 tests); values in *italics*: *P* < 0.01, underlined values *P <* 0.05.

### Community composition

**Fig 4:**
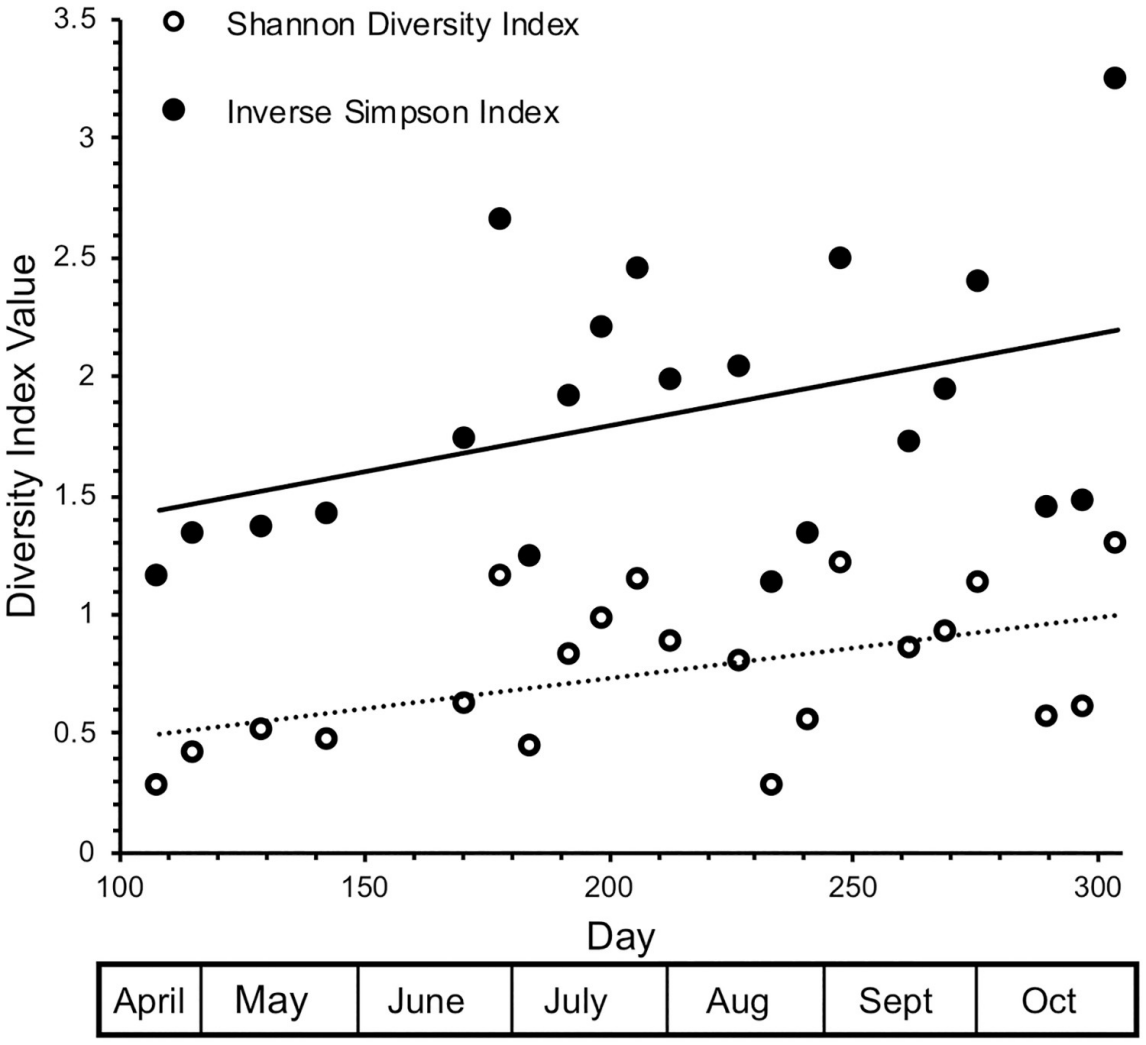
Diversity indices calculated by date. Both the Shannon Diversity index and the Inverse Simpson index increased between April and October.

**Fig 5:**
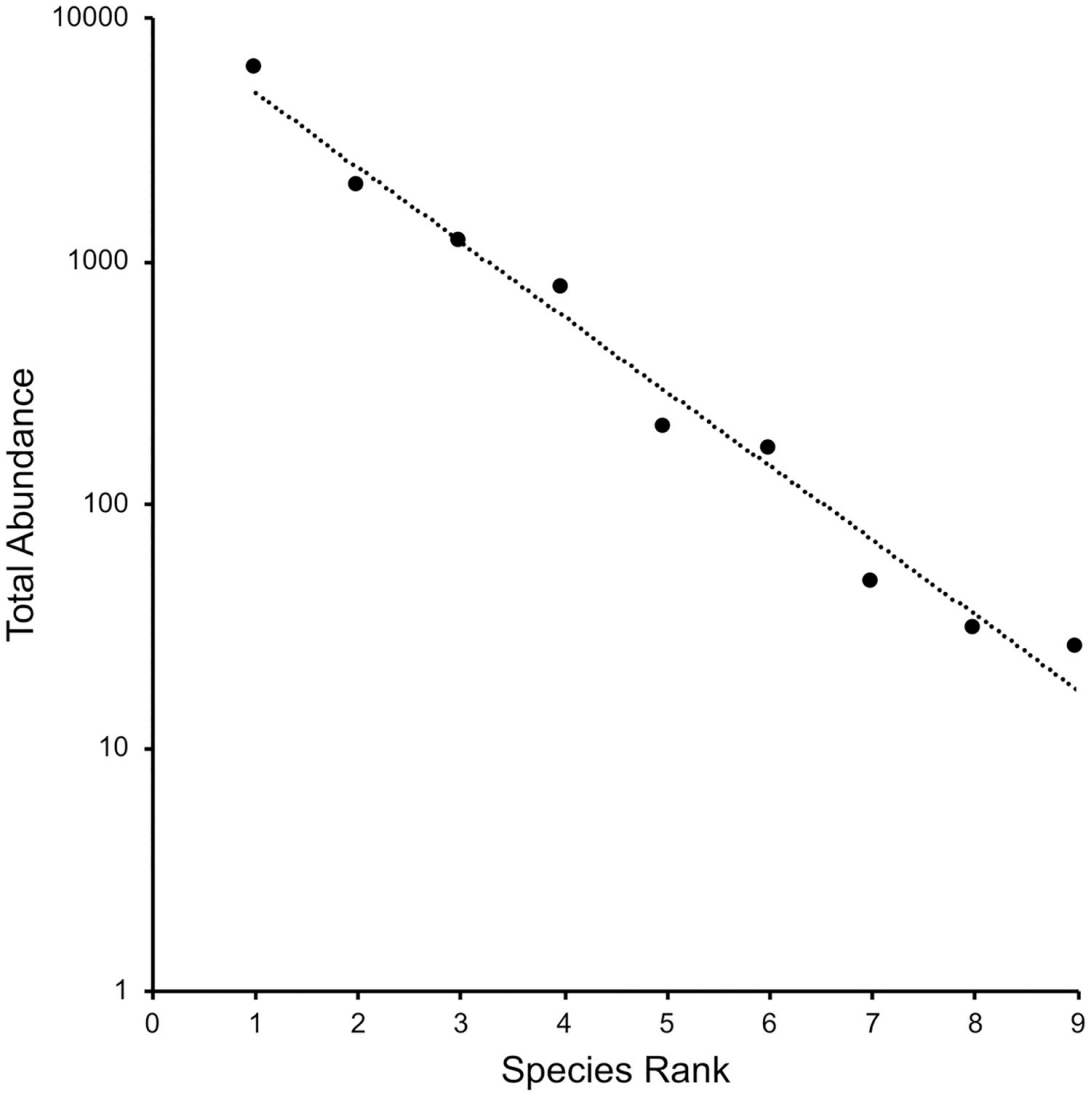
Rank abundance plot showing diversity of the nine species caught over the entire season. The relationship fits a log series model (r^2^ = 0.978, *P <<* 0.001). The species are, in order of rank, *D*. *simulans*, *D*. *melanogaster*, *D*. *suzukii*, *D*. *algonquin*, *D*. *hydei*, *D*. *affiniis*, *D*. *tripunctata*, *D*. *busckii* and *D*. *melanica*.

Collections later in the season were more diverse than those earlier in the season (S2 Table) as was reflected in the positive correlation between Shannon Diversity Index and date (Fig 4; r^2^ = 0.272, *P =* 0.015). The Inverse Simpson Index, a measure of dominance, showed an insignificant increase over the season (r^2^ = 0.177, *P =* 0.057). However, the abundance of the species fits a log series model (Fig 5, r^2^ = 0.978, *P <<* 0.001), a situation in which Simpson’s index is insensitive to species richness [47]. Overall, the diversity of the samples was low and variable across the season.

### Fluctuations in abundance and environmental variables

To understand the relationship between species abundance and temperature, maximum likelihood calculations were applied to the capture data for the four most abundant species. For each species, a succession of models was calculated using the parameters in the order φ_0_, A, B, C, and D (Eq 6). The addition of each parameter dramatically improved the log likelihood function until the parameter for humidity, D, was added (S5 Table). For all the species, humidity, when added to the model in the presence of temperature and temperature squared, did not contribute significantly to the explanation of the counts of captured flies. The failure of humidity to contribute to these models was probably because of the negative correlation between temperature and humidity (as the temperature rose, humidity declined during the day). The models, based on 21 observations, had significant r^2^ values, all *P* < 0.001 (Table 4).

**Table 4:** Best model parameter estimates for the four species^a^.

Species	φ_0_	Constant (A)	Temperature (B)	Temperature^2^ (C)	ln(L)	r^2^ ^b^
*D*. *algonquin*	3.656	0.0073	-0.00019	-0.00001	-387	0.704
*D*. *melanogaster*	0.280	-0.0665	0.00934	-0.00026	-536	0.822
*D*. *simulans*	0.045	-0.0693	0.00918	-0.00025	-2160	0.826
*D*. *suzukii*	0.281	-0.0736	0.00859	-0.00022	-366	0.748

^a^Letters with each parameter correspond to the equations in the Materials and Methods

^b^All r^2^ values are significant (*P* < 0.001)

Parameter estimates varied greatly among the four species (Table 4). The relatively large value of φ_0_, the relative size abundance at the start of the season, for *D*. *algonquin* reflected the early presence in the traps for this species. Although the first traps were put out in late April, relatively early for most species, *D*. *algonquin* already had a strong presence. *D*. *simulans*, in contrast, with a small value of φ_0_, was very late to appear in the traps.

The negative coefficients for temperature (B) and temperature squared (C) implied that, with the rising temperatures toward the middle of the summer, the *D*. *algonquin* population was declining until it had completely disappeared by July. The other species, by contrast, had positive coefficients for temperature. They all responded to rising temperatures through substantial population growth by late summer.

Because there was a negative coefficient to the squared term for temperature (C, Eq 6), we calculated the optimum temperature for growth implied by these models (Table 5), except for *D*. *algonquin*, which was already in decline in the early part of the study. In particular *D*. *suzukii* responded strongly to the warmer temperatures of the late summer. The models also allowed us to estimate the minimum and maximum temperatures for which the growth rate was positive (Table 5). For *D*. *melanogaster* the range of positive growth was between 9.9°C and 25.4°C. Outside of this range the population declined. For *D*. *simulans* the range was slightly larger and shifted to higher temperatures. For *D*. *suzukii* the range was smaller (12.5–27.2°C) but shifted to higher temperatures compared to the other species. In contrast, *D*. *algonquin* fell into decline at the relatively modest 17.4°C, which was closer to the optimum temperature for positive growth of the other species.

**Table 5:** Implications of the parameter estimates.

Species	Daylight Hours to One Fly per Hour^a^	Temperature of Maximum Growth (°C)	Minimum Temperature for Positive Growth (°C)	Maximum Temperature for Positive Growth (°C)
*D*. *algonquin*^b^				17.4
*D*. *melanogaster*	117	17.6	9.9	25.4
*D*. *simulans*	250	18.5	10.6	26.4
*D*. *suzukii*	120	19.8	12.5	27.2

^a^Assuming a constant temperature of 22°C and 70% relative humidity, this is the number of daylight hours required for the population to reach a size that would result in one capture per hour.

^b^Sampling of *D*. *algonquin* was insufficient to estimate most of the parameters

**Fig 6:**
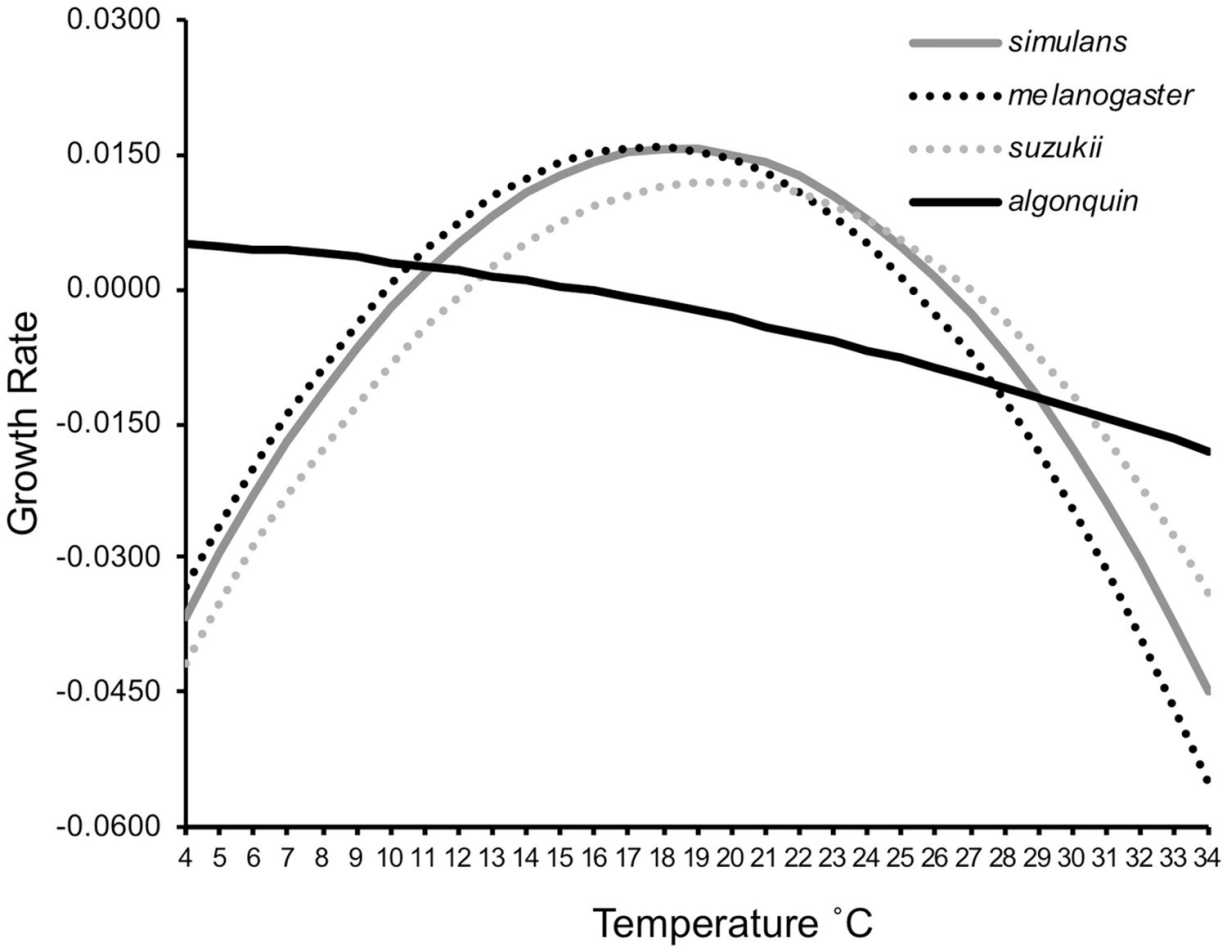
Growth rates at temperatures across the season inferred from the model. Using φ as calculated from the data, the population growth of each species in the temperature model is plotted.

Given that the temperatures obtained from the model for population limits did not fit with empirical lab results (e.g. [51– 53]), although we had a reasonable fit for the population abundance, we built a final model that included separate equations for population growth and fly activity level. For the population growth, higher order terms in temperature made no difference to the equations for r. As a consequence, for all species except *D*. *algonquin*, the populations grew more quickly as the temperature increased (Fig 6). For *D*. *algonquin*, the population fell as the temperature climbed.

## Discussion

### Species and community composition

The species composition in our seasonal sample is variable across the season and the composition of our collections was notably less diverse than that of Yoshimoto [33, 34]. This may be in part because our collections were narrower in geography and bait type than those of Yoshimoto [33]. Our bait type was not optimal for all species given that few mushroom breeders are attracted to banana [54] and some species are only found in particular habitats. For example, *D*. *americana* is found along rivers on sandbar and black willows [55, 56], but our sites were distant from both rivers and willows. Other missing species were individually less than 2% of the Yoshimoto collection, and collectively 7% (Table 1). Nonetheless, two of the rarest species of that collection were represented at modest numbers in our collection (e.g., *D*. *melanica* and *D*. *algonquin*).

One notably absent species that was present in Yoshimoto [34] is *D*. *athabasca*, which is not found as far south as it used to be, except in the Appalachian Mountains [57]. Although global climate change has possibly eliminated this species from the area, collections must be done further afield. Nonetheless, the species has not been found in our casual collections in previous years at the University of Kansas Field Station, whereas all the species found in our orchard traps were also present at the field station (Roy and Gleason, unpublished). Miller [58] argued that *D*. *athabasca* was never this far south and in many collections was misidentified *D*. *affinis* or *D*. *algonquin*, both of which were found in our collection.

In addition to the presence and absence of species in our collection versus Yoshimoto [34], the most abundant species has changed over the last 60 years. In the Yoshimoto collection, the proportion of *D*. *simulans* was only 0.16% (Table 1) but it was the most dominant species in our collection (57.97%, Table 1). At the same time, *D*. *melanogaster* has dropped from 83.12% to 18.93% (Table 1). Directly comparing these numbers to previous records needs to be done cautiously because of differences in collection methods; however, the expansion of *D*. *simulans* appears to be a general trend across time and not the result of confusing *D*. *melanogaster* with *D*. *simulans*. In the late 1940s *D*. *simulans* was less than 1% of June and July collections in St. Louis [59] whereas *D*. *simulans* was 13.5% of our June and July collections (although absent in June; S3 Table).

The invasive species, *Z*. *indianus*, was not found in our 2014 collection, which followed a particular cold winter. November 2013 through February 2014 had 349 more heating degree days than normal. In contrast, *Z*. *indianus* was collected in September and October 2015 in the same locations (Gleason *et al*. unpublished) following a winter (November 2014-February 2015) that had only 91 heating degree days more than normal. These two observations suggest that the species may reinvade after populations are reduced because of the response to cold stress. *Zaprionus indianus* is particularly sensitive to cold [60] but additional work on the phenology of the species is needed to determine the likelihood that it will become established in this region.

### Growth model

Our population growth models based on species abundance provide estimates of the temperature ranges where positive growth occurred. In some cases, these estimates are different from those estimated in the laboratory. Within laboratory studies, different labs have found different optimal temperatures and relevance to ecological conditions has been questioned. For example, *D*. *simulans* in the lab responded to cold better than *D*. *melanogaster* and performed more poorly in response to heat [53], yet this was the opposite of our observations of the species under ecological conditions (Table 5). Other studies have found a lower preferred temperature for *D*. *simulans* (20.5°C) versus *D*. *melanogaster* (21.3°C [51]). Ecophysiological responses to temperature are usually measured as stress response or developmental rate, but survivorship is only part of fitness. Reproductive fitness may be affected by temperature in a different manner; ovariole number in the lab peaks for *D*. *simulans* at a higher temperature (24°C) than it does for *D*. *melanogaster* (24°C [61]).

For *D*. *melanogaster*, viability has been estimated to occur between 10 and 32°C with fertility between 12 and 30°C; optimal temperatures are between 14 and 29°C (reviewed in [62]). Fecundity peaks in the lab at approximately 25°C [63], which is much higher than the estimated temperature for optimal growth rate (17.6°C, Table 5), and the maximum temperature for positive growth (25°C, Table 5) based on parameters estimated in our growth model. This is likely caused by the use of constant temperatures in the laboratory whereas our measurements are from a thermally variable environment. Thermal variation can have dramatic effects on many life history traits, including development time, fecundity, and stress tolerance [63–66]. In addition, the occurrence of extreme temperatures likely plays a significant role in population growth. In a lab temperature gradient experiment, with stepped temperatures from 10 to 25°C in 5°C increments, the optimum temperature for *D*. *melanogaster* was estimated to be 20.9°C [52], which is consistent with the estimates from our population growth models (Table 5).

Like *D*. *melanogaster*, *D*. *simulans* is plastic in temperature responses with different geographic lines responding optimally across a wide range of temperature between 14 and 30°C, regardless of origin [67]. Only copulation success is diminished at lower temperatures though males are persistent in courting [67]. In the same stepped temperature gradient experiment as for *D*. *melanogaster* [52], the optimum temperature for *D*. *simulans* was 19.0°C, which contrasts with our estimate of an optimal temperature for *D*. *simulans* that is higher than that of *D*. *melanogaster*.

Male and female *D*. *melanogaster* and *D*. *simulans* have different temperature tolerances [68]. For example, *D*. *melanogaster* females are behaviorally more sensitive to heat than are males [69]. Because we cannot distinguish the females of these two species, we cannot directly address how sex affects population abundance in our fly traps. A high throughput diagnostic test would aid in understanding sex effects in abundance.

In our models, *D*. *suzukii* had the highest estimated temperature for maximum growth (27.2°C, Table 5). For this species, lab estimates put an upper threshold for survivorship at 31°C [70] whereas field observations indicate that adults of the species are not found in traps when the temperature average is greater than 28°C or is above 33°C for more than 8 hours [71]. This contrasts with an egg-to-adult developmental optimum of 28.2°C [72], though highest fecundity is at 22.8°C [72], indicating that thermal tolerance is dependent on life stage. The minimum threshold average daily temperature for development under constant conditions has been estimated to be 7.2°C [70] or 8.1°C [72] though others have suggested the species is unlikely to survive below 10°C [73] or even at 11.6°C with variable conditions [74]. Low temperature tolerance changes with morphology as those with a dark morphology have a lower temp resistance than light morphs [75]. The dark morph is induced by low temperatures, independent of day length, and is accompanied by increased body size and reproductive quiescence, which likely collectively contribute to increased thermal stress resistance [76–79].

### Phenology of *D*. *melanogaster* and *D*. *simulans*

Few studies have examined the abundance of these species with monthly sampling and almost none as frequently as our samples. Still, the pattern in abundance in our collections, in which *D*. *melanogaster* appears earlier in the growing season than *D*. *simulans*, has been observed in many other temperate locations in both the Northern and Southern hemisphere (e.g. [19, 21, 29, 80, 81]). Temperature is a likely driving force for the difference in species presence in the collections. In Brazil, *D*. *melanogaster* is negatively correlated with high temperatures (above 29°C) and low temperatures (below 12°C) indicating that mid temperature ranges are ideal [16]. The abundance of *D*. *simulans*, which had a population 30 times that of *D*. *melanogaster* in Brazil, is not correlated with temperature. Thus, *D*. *simulans* may have faster population growth when it is hotter, but are slow to get started when winters are cold, as in the temperate region. *Drosophila melanogaster* is more cold resistant and behaviorally more likely to survive overwintering at higher numbers because the species will enter buildings whereas *D*. *simulans* will not [82]. This implies that the severity of cold temperatures in the winter should affect the date at which *D*. *simulans* becomes abundant during the summer. The two species have additional differences in life span and under-feeding tolerance that gives *D*. *simulans* a wider performance breadth, but makes it less resistant at colder extremes in the lab [51, 81].

Humidity has been proposed as a factor in the abundance of these species. In the lab, *D*. *melanogaster* is more desiccation resistant than *D*. *simulans* [83] but these measures were taken at 0% relative humidity and do not reflect growth in variable humidity environments. The absence of an effect of humidity in our study probably reflects that humidity is rarely low during the growing season in Kansas, and is continuously correlated with temperature, thus temperature has a larger effect than humidity on growth rates.

### Phenology of *D*. *suzukii*

The phenology of *D*. *suzukii* is similar to *D*. *simulans* in that it appears in appreciable numbers in July and peaks in October (S1 Fig), though it is the dominant species at the end of August (Fig 3). Other localities have found it to be a late season species: infestation on blueberries in Rhode Island peaked in August [84]; *D*. *suzukii* in Tuscany, Italy, are present in June and July but peak in August [85]; in the Willamette Valley of Oregon where *D*. *suzukii* is found on cherries, early peak abundances occur in July [70]. In contrast, near Winters, CA, where average winter temperatures are not below freezing and summers are hot and dry, peaks in abundance are found in spring and autumn [86], a pattern also seen in warmer areas of Spain [87]. Host switching occurs regularly with *D*. *suzukii* found on different fruits as they ripen [88]. Together this evidence indicates that *D*. *suzukii* may differ in phenology in different climatic regions.

The similarity we see here between *D*. *simulans* and *D*. *suzukii* peak abundance is in contrast to populations in Southern Italy, where the two species were abundant at different times of the year, although Southern Italy has winter daily temperatures well above freezing [89]. In this location, hot dry weather coincides with the absence of *D*. *suzukii*. The pattern is repeated in the San Joaquin Valley, California, where abundance is locally high by fruit type in November through June, but the flies are not attracted to bait in July through October [90].

The number of individuals caught can be influenced by farm type. All of the flies in this study came from conventional orchards, but in one study examining *D*. *suzukii* associated with raspberries found that organic farms had far fewer *D*. *suzukii* than conventional farms [91]. Our original plan was to collect *Drosophila* at two conventional orchards and at least one organic farm; however, we caught very few *Drosophila* at the organic farm sites and we chose to not continue the collections after mid-summer when the other farms had a variety of flies, thus anecdotally we confirm the pattern observed.

Although recently invasive, *D*. *suzukii* has the potential to become a dominant species in Kansas given that is was 11.22% of the total collection (Table 1). Able to exploit ripening fruit, whereas other species are restricted to rotting fruit, *D*. *suzukii* may reduce its competition with other species through use of alternate larval hosts, thus predicting whether or not it will displace native species is difficult. Together with its ability to exploit non-fruit species [92], *D*. *suzukii* has the colonizing ability of *D*. *melanogaster* and *D*. *simulans* and is likely to be locally established near humans.

### Phenology of other species

The native species, *D*. *algonquin* was one of the earliest detected species, already present when we started our collections in April. To fully understand the phenology of *D*. *algonquin* will require starting collections earlier in the season. *D*. *algonquin* disappeared from collections in the latter half of the collecting season, thus it never overlapped with *D*. *affinis*, its closest relative in the collection. Other collections of these species had the same pattern in that *D*. *algonquin* is the predominate species in colder months and is replaced by *D*. *affinis* in warmer months [22, 93, 94]. In lab cultures, *D*. *affinis* outcompetes *D*. *algonquin* at higher temperatures [22], and has greater heat tolerance [94], indicating that these species are adapted to different seasonal temperatures resulting in the observed seasonal isolation.

Appearing early and then again late in the season implies that *D*. *hydei* is a cooler weather species. Its abundance was not sufficient to allow closer examination of its relationship with temperature. The other native species were rare and similarly it is hard to make inferences about their phenological patterns. *Drosophila tripunctata*, a mycophagous species with low desiccation resistance [95] and low survivorship at 30°C [96], was only found in the late part of the collecting season, as temperatures decreased.

## Conclusion

Our data demonstrate that species differ in phenology in Kansas in analogous ways to that at other locations around the world for Cosmopolitan species. Additional long-term data is needed to understand fluctuations in *Drosophila* populations from year to year, particularly to test the implications of the models developed here, which may have implications for the response of species to climate change. Basic ecology and physiology of multiple insects are needed for studies on the effect of climate change in insects, which has been dominated by pest species of agriculture and forests [97]. *Drosophila* offer an ideal species collection with variable phylogenetic relationships to allow tests of adaptation. We agree with previous authors that a community based analysis to understand the dynamics of all species will improve our understanding of the effect of pest species (e.g. *D*. *suzukii* [14]). *Drosophila* assemblages are good bioindicators of climate change [98] and habitat fragmentation and urbanization [27, 99].

## Supporting information

S1 Fig Abundance of each species in each collection corrected for the number of days of the collection. Note that the Y-axis scale varies among the graphs. A. The five least abundant species. B. Three of the most abundant species, *D*. *algonquin*, *D*. *melanogaster* and *D*. *suzukii*. C. The most abundant species, *D*. *simulans*.
(PDF)

S1 Table Fruit grown at the collection locations. (DOCX)

S2 Table Summary statistics for collection dates. (DOCX)

S3 Table Proportion of each collection represented by each species. (DOCX)

S4 Table Summary statistics for each species. (DOCX)

S5 Table Growth parameter estimates. (DOCX)
