# Supplementary material for: Phenology of Drosophila species across a temperate growing season and implications for behavior
Source: PLoS One. 2019 May 16;14(5):e0216601. doi: 10.1371/journal.pone.0216601 (PMC6521991; doi:10.1371/journal.pone.0216601)
Supplement: S1 Fig — Note that the Y-axis scale varies among the graphs. A. The five least abundant species. B. Three of the most abundant species, D. algonquin, D. melanogaster and D. suzukii. C. The most abundant species, D. simulans. (PDF) [file pone.0216601.s001.pdf]

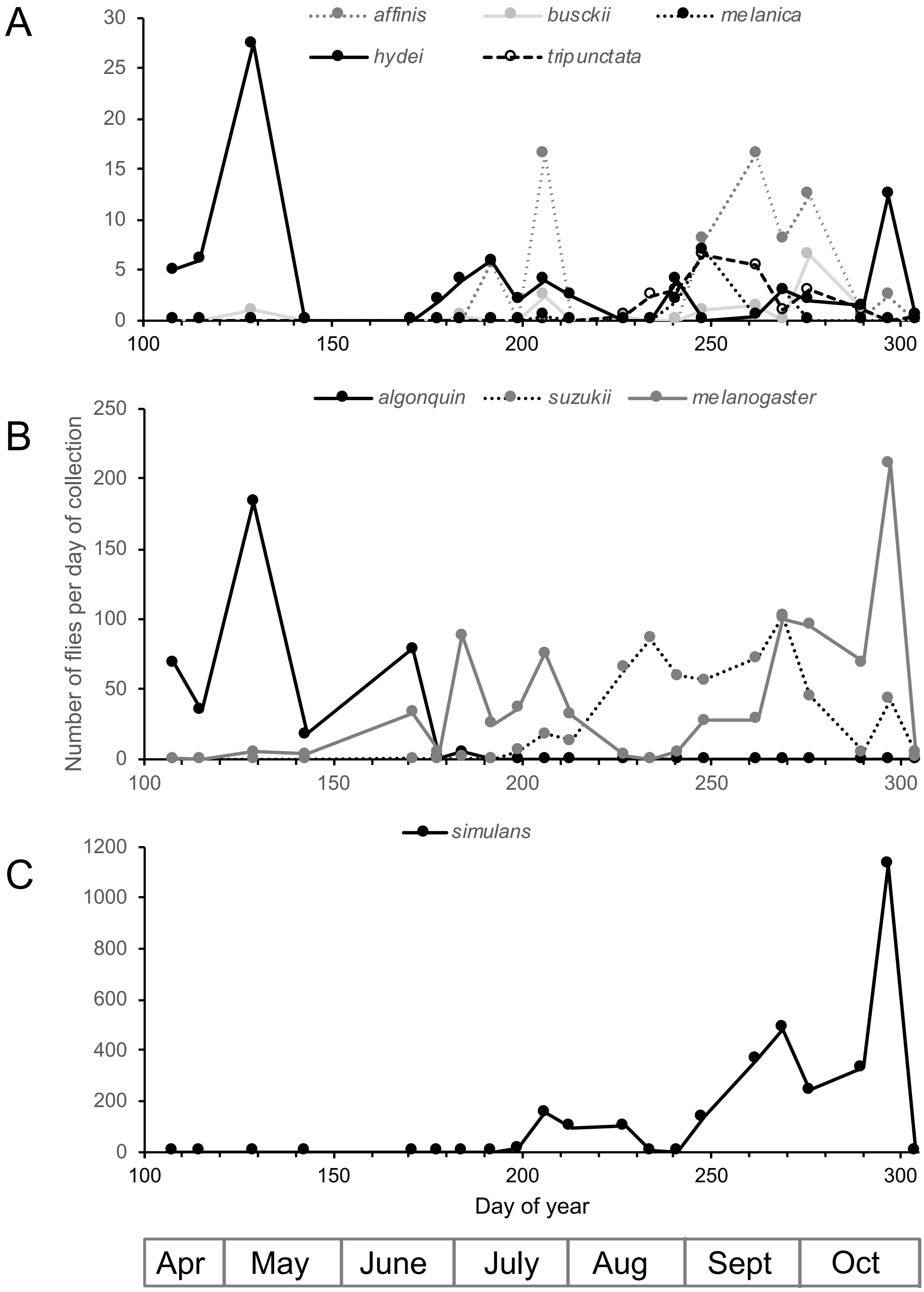

S1 Fig. Abundance of each species in each collection corrected for the number of days of the collection. Note that the Y-axis scale varies among the graphs. A. The five least abundant species. B. Three of the most abundant species, *D. algonquin, D. melanogaster* and *D. suzukii*. C. The most abundance species, *D. simulans*.
