# Supplementary material for: Phenology of Drosophila species across a temperate growing season and implications for behavior
Source: PLoS One. 2019 May 16;14(5):e0216601. doi: 10.1371/journal.pone.0216601 (PMC6521991; doi:10.1371/journal.pone.0216601)
Supplement: S1 Table — (DOCX) [file pone.0216601.s002.docx]

**S1 Table. Fruit grown at the collection locations**

| Fruit | Rees’ Fruit Farm | 86^th^ Street Orchard |
| --- | --- | --- |
| Strawberries | X |  |
| Gooseberries | X |  |
| Red Raspberries | X |  |
| Black Raspberries | X |  |
| Tart Cherries | X |  |
| Blackberries | X | X |
| Peaches | X |  |
| Nectarines | X |  |
| Apples | X | X |
| Grapes | X | X |
| Pears |  | X |
