# Supplementary material for: Phenology of Drosophila species across a temperate growing season and implications for behavior
Source: PLoS One. 2019 May 16;14(5):e0216601. doi: 10.1371/journal.pone.0216601 (PMC6521991; doi:10.1371/journal.pone.0216601)
Supplement: S2 Table — (DOCX) [file pone.0216601.s003.docx]

**S2 Table. Summary statistics for collection dates**

| First day of collection (2014) | Day of year | Number of days of the collection | Number of individuals^a^ | Richness | Shannon diversity index | Simpson inverse index | Evenness^b^ | Evenness corrected for all species^b^ |
| --- | --- | --- | --- | --- | --- | --- | --- | --- |
| April 18 | 108 | 2 | 147 | 3 | 0.289 | 1.162 | 0.263 | 0.131 |
| April 25 | 115 | 2 | 81 | 2 | 0.419 | 1.338 | 0.605 | 0.191 |
| May 9 | 129 | 2 | 434 | 4 | 0.515 | 1.367 | 0.372 | 0.234 |
| May 23 | 143 | 2 | 44 | 2 | 0.474 | 1.424 | 0.684 | 0.216 |
| June 20 | 171 | 2 | 225 | 3 | 0.636 | 1.737 | 0.579 | 0.289 |
| June 27 | 178 | 2 | 20 | 4 | 1.116 | 2.667 | 0.841 | 0.530 |
| July 3 | 184 | 2 | 197 | 5 | 0.456 | 1.246 | 0.283 | 0.207 |
| July 11 | 192 | 7 | 259 | 3 | 0.838 | 1.919 | 0.763 | 0.381 |
| July 18 | 199 | 7 | 418 | 4 | 0.992 | 2.209 | 0.715 | 0.451 |
| July 25 | 206 | 2 | 543 | 7 | 1.148 | 2.461 | 0.590 | 0.522 |
| August 1 | 213 | 2 | 286 | 4 | 0.895 | 1.998 | 0.646 | 0.407 |
| August 15 | 227 | 2 | 344 | 5 | 0.810 | 2.039 | 0.503 | 0.369 |
| August 22 | 234 | 2 | 184 | 3 | 0.285 | 1.142 | 0.260 | 0.130 |
| August 29 | 241 | 2 | 139 | 4 | 0.560 | 1.350 | 0.404 | 0.255 |
| September 5 | 248 | 2 | 484 | 7 | 1.218 | 2.503 | 0.626 | 0.554 |
| September 19 | 262 | 2 | 974 | 8 | 0.867 | 1.727 | 0.417 | 0.394 |
| September 26 | 269 | 2 | 1405 | 7 | 0.935 | 1.944 | 0.480 | 0.425 |
| October 3 | 276 | 2 | 813 | 7 | 1.137 | 2.401 | 0.584 | 0.517 |
| October 17 | 290 | 2 | 817 | 7 | 0.559 | 1.455 | 0.298 | 0.263 |
| October 24 | 297 | 2 | 2800 | 5 | 0.620 | 1.477 | 0.385 | 0.282 |
| October 31 | 304 | 2 | 24 | 5 | 1.310 | 3.256 | 0.814 | 0.596 |
| Mean^c^ |  | 2.476 | 506.571 | 4.714 | 0.769 | 1.848 | 0.529 | 0.350 |
| Standard deviation^c^ |  | 1.468 | 618.575 | 1.777 | 0.308 | 0.558 | 0.178 | 0.140 |
| Total data set^d^ |  | 52 | 10638 | 9 | 1.263 | 2.547 | 0.575 | 0.575 |

^a^ Data are not corrected for number of collection days.

^b^Evenness was calculated for collection using the number of species in that particular collection and for all nine species, thereby taking into account the species that were missing in a collection.

^c^Mean and standard deviations were calculated across all dates.

^d^Total data set calculations were obtained by combining all dates.
