# Supplementary material for: Phenology of Drosophila species across a temperate growing season and implications for behavior
Source: PLoS One. 2019 May 16;14(5):e0216601. doi: 10.1371/journal.pone.0216601 (PMC6521991; doi:10.1371/journal.pone.0216601)
Supplement: S3 Table — (DOCX) [file pone.0216601.s004.docx]

**S3 Table. Proportion of each collection represented by each species**

| First day of collection ^a^ | *D. algonquin* | *D. affinis* | *D. busckii* | *D. hydei* | *D. melanica* | *D. melanogaster* ^b^ | *D. simulans* ^b^ | *D. suzukii* | *D. tripunctata* |
| --- | --- | --- | --- | --- | --- | --- | --- | --- | --- |
| April 18 | 0.925 | 0 | 0 | 0.068 | 0 | 0.007 | 0 | 0 | 0 |
| April 25 | 0.852 | 0 | 0 | 0.148 | 0 | 0 | 0 | 0 | 0 |
| May 9 | 0.846 | 0 | 0.005 | 0.127 | 0 | 0.023 | 0 | 0 | 0 |
| May 23 | 0.818 | 0 | 0 | 0 | 0 | 0.182 | 0 | 0 | 0 |
| June 20 | 0.698 | 0 | 0 | 0 | 0 | 0.298 | 0 | 0.004 | 0 |
| June 27 | 0.150 | 0 | 0.100 | 0.200 | 0 | 0.550 | 0 | 0 | 0 |
| July 3 | 0.051 | 0 | 0.005 | 0.041 | 0 | 0.893 | 0 | 0.010 | 0 |
| July 11 | 0 | 0.154 | 0 | 0.158 | 0 | 0.687 | 0 | 0 | 0 |
| July 18 | 0 | 0 | 0 | 0.033 | 0 | 0.616 | 0.247 | 0.103 | 0 |
| July 25 | 0 | 0.061 | 0.009 | 0.015 | 0.002 | 0.284 | 0.563 | 0.066 | 0 |
| August 1 | 0 | 0 | 0 | 0.017 | 0 | 0.226 | 0.666 | 0.091 | 0 |
| August 15 | 0 | 0 | 0.003 | 0 | 0 | 0.026 | 0.587 | 0.381 | 0.003 |
| August 22 | 0 | 0 | 0 | 0 | 0 | 0 | 0.038 | 0.935 | 0.027 |
| August 29 | 0 | 0 | 0 | 0 | 0.029 | 0.072 | 0 | 0.856 | 0.043 |
| September 5 | 0 | 0.033 | 0.004 | 0 | 0.029 | 0.098 | 0.578 | 0.231 | 0.027 |
| September 19 | 0 | 0.034 | 0.003 | 0.001 | 0.001 | 0.059 | 0.743 | 0.148 | 0.011 |
| September 26 | 0 | 0.011 | 0 | 0.007 | 0.004 | 0.143 | 0.688 | 0.145 | 0.001 |
| October 3 | 0 | 0.031 | 0016 | 0.005 | 0 | 0.245 | 0.586 | 0.111 | 0.007 |
| October 17 | 0 | 0.001 | 0.002 | 0.004 | 0 | 0.166 | 0.812 | 0.012 | 0.002 |
| October 24 | 0 | 0.002 | 0 | 0.010 | 0 | 0.149 | 0.809 | 0.030 | 0 |
| October 31 | 0 | 0 | 0 | 0.042 | 0 | 0.081 | 0.361 | 0.375 | 0.042 |

^a^ See S2 Table for the day of year and number of days of each collection

^b^ Because female *D. melanogaster* and *D. simulans* are not distinguishable, the total females collected for these species were distributed to the species in the same proportion as the males of the species in the collection.
