# Supplementary material for: Phenology of Drosophila species across a temperate growing season and implications for behavior
Source: PLoS One. 2019 May 16;14(5):e0216601. doi: 10.1371/journal.pone.0216601 (PMC6521991; doi:10.1371/journal.pone.0216601)
Supplement: S4 Table — (DOCX) [file pone.0216601.s005.docx]

**S4 Table. Summary statistics for each species**

| Species | Number of | | | | | | | | Proportion male^a^ | | Number of collections^b^ | | | | | | Mean number individuals per day  in a collection^c^ | | | | |
| --- | --- | --- | --- | --- | --- | --- | --- | --- | --- | --- | --- | --- | --- | --- | --- | --- | --- | --- | --- | --- | --- |
|  | | | individuals | | females | | males | | |  | |  | | | All | | | Present | | |  |
| *affinis* | | 169 | | 69 | | 100 | | 0.59 | | | | | | 9 | 3.34 ± 5.63 | | | | | 7.80 ± 6.33 | |
| *algonquin* | | 778 | | 329 | | 449 | | **0.58** | | | | | | 7 | 18.53 ± 44.04 | | | | | 55.57 ± 63.74 | |
| *busckii* | | 31 | | 20 | | 11 | | 0.35 | | | | | | 9 | 0.73 ± 1.48 | | | | | 1.72 ± 1.89 | |
| *hydei* | | 205 | | 137 | | 68 | | **0.33** | | | | | | 15 | 3.95 ± 6.38 | | | | | 5.52 ± 6.98 | |
| *melanica* | | 26 | | 17 | | 9 | | 0.35 | | | | | | 5 | 0.61 ± 1.65 | | | | | 2.60 ± 2.68 | |
| *suzukii* | | 1184 | | 668 | | 516 | | **0.44** | | | | | | 15 | 27.46 ± 33.56 | | | | | 38.44 ± 34.01 | |
| *tripunctata* | | 47 | | 14 | | **33** | | *0.70* | | | | | | 9 | 1.12 ± 1.91 | | | | | 2.61 ± 2.18 | |
| *melanogaster* and *simulans*^d^ | | 8198 | | 4573 | | 3625 | | **0.44** | | | | | | 21 | 186.02 ± 312.73 | | | | | 195.33 ± 317.86 | |
| Total | | 10584 | | 5827 | | 4811 | | **0.45** | | | | | 21 | | | 241.77 ± 321.95 | | |  | | |
| *melanogaster* males | | 939 | |  | |  | |  | | | | | 19 | | | 17.80 ± 2.18 | | | 19.68 ± 22.53 | | |
| *simulans* males | | 2686 | |  | |  | |  | | | | | 12 | | | 63.02 ± 116.82 | | | 110.28 ± 138.30 | | |

^a^Chi-squared test comparison to 1:1 sex ratio, ***P*<<0.001**, *P<0.01*, *P*<0.05.

^b^The total number of collections are presented in which each species is found. The total reflects the total number of collections across the whole season: all had at least one fly present.

^c^Collections were made over either 2 or 7 days. The number of individuals collected was corrected for the number of days of the collection. Data presented are mean ± standard deviation*.* The data are presented for all collections and only those collections in which the species is found.

^d^*D. melanogaster* and *D. simulans* were grouped together because females for the two species were indistinguishable. Separate data for the males are presented below the totals for all species.
