# Supplementary material for: Phenology of Drosophila species across a temperate growing season and implications for behavior
Source: PLoS One. 2019 May 16;14(5):e0216601. doi: 10.1371/journal.pone.0216601 (PMC6521991; doi:10.1371/journal.pone.0216601)
Supplement: S5 Table — (DOCX) [file pone.0216601.s006.docx]

**S5 Table. Growth Parameter Estimates**

1. ***D. algonquin***

| Model | λ_0_ | Constant | Temperature | Temperature Squared | Humidity | ln(L) |
| --- | --- | --- | --- | --- | --- | --- |
| 1 | 1.12 |  |  |  |  | -1415 |
| 2 | 12.97 | -0.0025 |  |  |  | -543 |
| 3 | 4.43 | 0.0079 | -0.00051 |  |  | -410 |
| 4 | 13.45 | -0.0031 | 0.00000 |  | 0.000007 | -548 |
| 5 | 3.70 | 0.0073 | -0.00019 | -0.00001 |  | -387 |
| 6 | 3.54 | 0.0056 | -0.00008 | -0.00002 | 0.000025 | -381 |

1. ***D. melanogaster****

| Model | λ_0_ | Constant | Temperature | Temperature Squared | Humidity | ln(L) |
| --- | --- | --- | --- | --- | --- | --- |
| 1 | 2.89 |  |  |  |  | -1317 |
| 2 | 0.49 | 0.0010 |  |  |  | -862 |
| 3 | 0.65 | -0.0017 | 0.00013 |  |  | -941 |
| 4 | 1.13 | -0.0067 |  |  | 0.0001020 | -821 |
| 5 | 0.28 | -0.0665 | 0.00935 | -0.00027 |  | -531 |
| 6 | 0.31 | -0.0665 | 0.00937 | -0.00027 | -0.0000004 | -535 |

1. ***D. simulans****

| Model | λ_0_ | Constant | Temperature | Temperature Squared | Humidity | ln(L) |
| --- | --- | --- | --- | --- | --- | --- |
| 1 | 8.92 |  |  |  |  | -8876 |
| 2 | 0.05 | 0.0025 |  |  |  | -3073 |
| 3 | 0.04 | 0.0025 | 0.00001 |  |  | -3058 |
| 4 | 0.13 | -0.0050 | 0.00000 |  | 0.000099 | -3038 |
| 5 | 0.04 | -0.0693 | 0.00921 | -0.00025 |  | -2161 |
| 6 | 0.04 | -0.0695 | 0.00925 | -0.00025 | 0.000016 | -2298 |

1. ***D. suzukii***

| Model | λ_0_ | Constant | Temperature | Temperature Squared | Humidity | ln(L) |
| --- | --- | --- | --- | --- | --- | --- |
| 1 | 1.71 |  |  |  |  | -1143 |
| 2 | 0.09 | 0.0016 |  |  |  | -658 |
| 3 | 0.11 | -0.0035 | 0.00024 |  |  | -432 |
| 4 | 0.27 | -0.0059 |  |  | 0.000097 | -747 |
| 5 | 0.24 | -0.0729 | 0.00866 | -0.00022 |  | -413 |
| 6 | 0.22 | -0.0728 | 0.00865 | -0.00022 | 0.000001 | -411 |

*Female *D. melanogaster* and *D. simulans* were indistinguishable. Females were apportioned to each species in the same ratio as the males.
